# Reward Contexts Extend Dopamine Signals to Unrewarded Stimuli

**DOI:** 10.1016/j.cub.2013.10.061

**Published:** 2014-01-06

**Authors:** Shunsuke Kobayashi, Wolfram Schultz

**Affiliations:** 1 Department of Physiology, Development and Neuroscience, University of Cambridge, Cambridge CB2 3DY, UK

## Abstract

Basic tenets of sensory processing emphasize the importance of accurate identification and discrimination of environmental objects [1]. Although this principle holds also for reward, the crucial acquisition of reward for survival would be aided by the capacity to detect objects whose rewarding properties may not be immediately apparent. Animal learning theory conceptualizes how unrewarded stimuli induce behavioral reactions in rewarded contexts due to pseudoconditioning and higher-order context conditioning [2, 3, 4, 5, 6]. We hypothesized that the underlying mechanisms may involve context-sensitive reward neurons. We studied short-latency activations of dopamine neurons to unrewarded, physically salient stimuli while systematically changing reward context. Dopamine neurons showed substantial activations to unrewarded stimuli and their conditioned stimuli in highly rewarded contexts. The activations decreased and often disappeared entirely with stepwise separation from rewarded contexts. The influence of reward context suggests that dopamine neurons respond to real and potential reward. The influence of reward context is compatible with the reward nature of phasic dopamine responses. The responses may facilitate rapid, default initiation of behavioral reactions in environments usually containing reward. Agents would encounter more and miss less reward, resulting in survival advantage and enhanced evolutionary fitness.

## Highlights

• Dopamine neurons are activated by unrewarded events in rewarded contexts
• More rewarded contexts are associated with stronger dopamine activations
• The effective unrewarded events do not induce bidirectional prediction error signals
• Reward context-dependent signaling conceivably leads to more reward

## Teaser

When rewards are frequent and everywhere, any object could be a reward. Reacting to objects in such reward contexts would enhance the chance of getting a reward. Kobayashi and Schultz show that dopamine neurons respond in reward contexts even to unrewarded objects.

## Results

Environmental contexts and situations exert strong influences on the interpretation of explicit events. Imagine yourself in a bomb shelter and hearing a loud bang. Then imagine sitting on a beach and hearing the same bang. Obviously, the behavioral reaction to the bang differs depending on the context, suggesting that information from the context (shelter versus beach) affects the interpretation of the explicit event (bang). Animal learning theory considers pseudoconditioning and higher-order conditioning to contextual cues as ways to conceptualize the influence of context on generating behavioral reactions. Pavlov noted “a conditioned reflex to the environment” [7], and Konorski mentioned “conditioned to situational cues” [8]. Thus, a primary reinforcer confers motivational value to contextual background via Pavlovian conditioning, and the motivational value then “spills over” to the explicit stimuli occurring in this background. In this way, an otherwise ineffective stimulus induces behavioral responses corresponding to the nature of the background [2, 6, 9], even though these events have never been paired with the primary reinforcer. In a rare mammalian neuronal study, auditory responses in bat inferior colliculus show wider tuning following aversive pseudoconditioning [10]. With reward, pseudoconditioning elicits approach behavior to unconditioned stimuli in rewarded contexts [3, 4, 5], as if the behavior generalizes to these stimuli. Through these fundamental phenomena, agents can associate stimuli with the reward characteristics of their environments without requiring more extensive explicit conditioning. The mechanism is advantageous when competing for limited resources and thus is evolutionarily adaptive. We tested possible underlying neuronal mechanisms in a prime reward system of the brain, the midbrain dopamine neurons. By systematically varying reward context, we demonstrate how dopamine neurons come to respond to unrewarded stimuli.

### Experimental Design and Behavior

Macaque monkeys viewed in six trial types three temporally unpredicted unconditioned stimuli (US) and three Pavlovian conditioned visual stimuli (CS) predicting, respectively, the three USs. The USs were (1) juice drops, (2) a large, salient, intensely colored abstract picture covering the computer monitor in front of them, and (3) a small abstract picture. The picture USs remained unchanged throughout thousands of trials before and during neuronal recordings and were never explicitly paired with reward before and during neuronal recordings. Novel stimuli were not tested.

**Figure 1:**
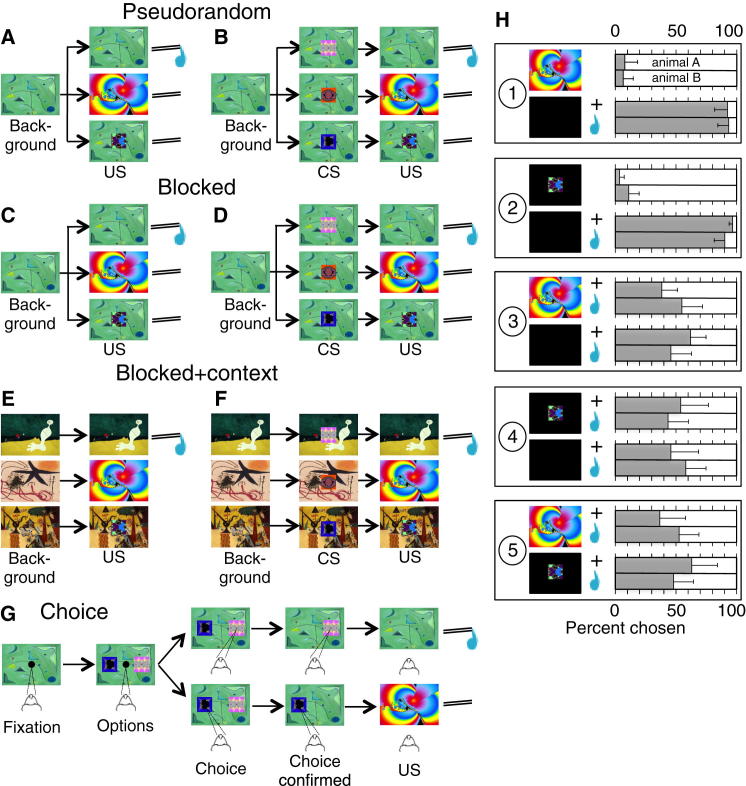
Stimuli and Behavior (A) “Pseudorandom” trials with three unpredicted unconditioned stimuli (USs), namely juice drops, large abstract picture, and small abstract picture (top to bottom). In this and all other figures, the small picture is not drawn to scale relative to the large picture. (B) Pseudorandom trials with three Pavlovian conditioned stimuli (CSs) predicting specifically the three USs. All US and CS trials alternated pseudorandomly in the presence of the same green background picture during all trial and intertrial periods. In this and all other figures, the CSs are not drawn to scale relative to the large US picture. (C and D) “Blocked” trials. The same three unpredicted USs (C), CSs (D), and background picture as in (A) and (B) were presented in separate blocks of 10–20 trials each. (E and F) “Blocked + context” trials with the same three unpredicted USs and CSs as in (A) and (B). In addition to trial blocks, each outcome was presented against a different background picture during all trial and intertrial periods, and the juice spout was inaccessible in large and small picture trials. (G) Ocular choice task, using CSs predicting juice or pictures. Example trial shows choice of juice (top) or small picture (bottom). (H) Behavioral preferences for juice and pictures in the ocular choice task. In panels 1–5, the USs of the two choice options are shown at top and bottom (note that choices were made between their respective CSs). Horizontal bars show choice frequencies (top, animal A; bottom, animal B); error bars show SD. Choice problems between respective CSs were as follows: (1) large picture versus black background + juice, (2) small picture versus black background + juice, (3) large picture + juice versus black background + juice, (4) small picture + juice versus black background + juice, (5) large picture + juice versus small picture + juice. Choice problems 1 and 2 alternated pseudorandomly (50 and 90 trials for each problem in animals A and B, respectively). Choice problems 3–5 alternated pseudorandomly (110–210 trials for each problem per animal). The identical juice reward in problems 3–5 served to maintain the animals’ motivation.

Three different contexts increasingly separated rewarded from unrewarded outcomes. In “pseudorandom” trials, the six trial types alternated pseudorandomly (Figures 1A and 1B). The juice, all picture USs, and all CSs occurred against the same background picture. Thus, juice reward occurred in 33% of trials, thus defining a single, common rewarded context. In “blocked” trials, each of the six trial types occurred in separate blocks of 10–20 identical trials against the common background picture (Figures 1C and 1D), with pseudorandom block alternation. Thus, rewarded and unrewarded trials constituted better-separated contexts. In “blocked + context” trials, the six trial types were also blocked. In addition, juice, large picture, and small picture occurred against their own separate background picture during all trial and intertrial periods, and the juice spout was removed with large and small pictures (Figures 1E and 1F). The animals noticed spout removal with mild body gestures. These blocks provided maximal context separation.

Because pictures may constitute genuine reward for monkeys [11, 12], we assessed their potential reward value with binary ocular choices between their respective CSs (Figure 1G). The animals preferred juice over any picture in >90% of trials, suggesting minor reward value of the pictures (Figures 1H1 and 1H2). To detect more subtle value differences, we tested each picture against the black monitor background. As the animals often refused task performance for the pictures alone, we added identical juice to both options (preventing reward conditioning by testing after all neuronal recordings were terminated). The animals were indifferent between each picture and the black background (H3, H4) and between small and large pictures (H5), suggesting that the pictures failed to add reward value to the juice. Similar repeatedly presented pictures have little own value [12], but complete absence of reward value is difficult to ascertain, as more interesting visual stimuli such as changing pictures, movies, and laboratory environments are known to be rewarding [12, 13].

### Neuronal Responses to Unpredicted Unconditioned Stimuli

**Figure 2:**
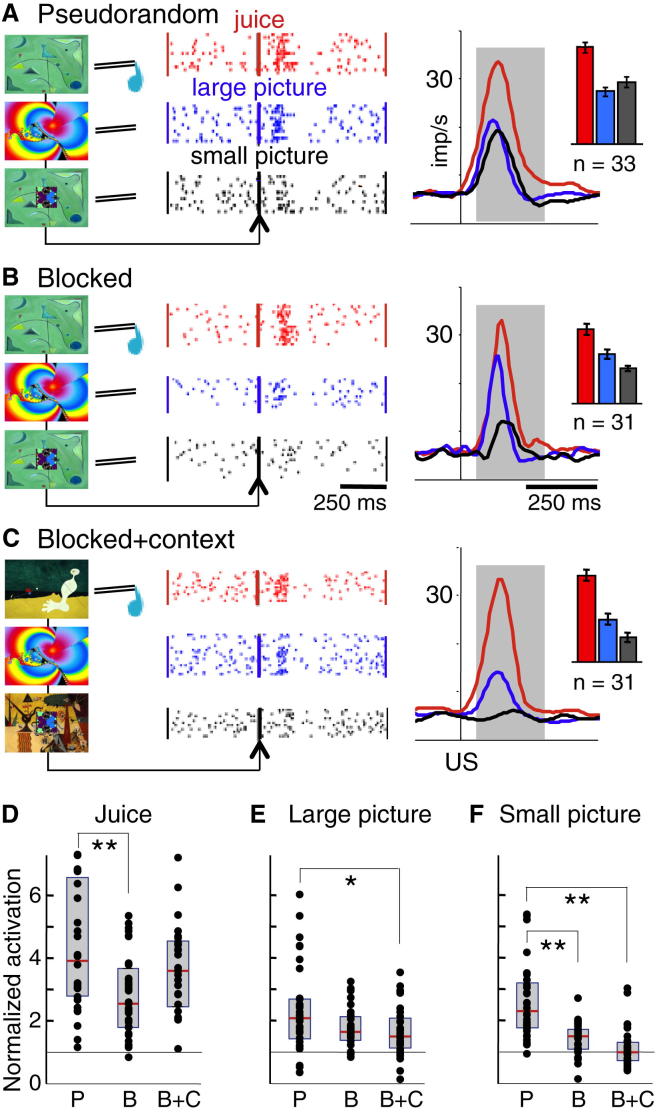
Effects of Context Separation on Dopamine Responses to Unpredicted Unconditioned Stimuli (A) Pseudorandom trials. Neuronal responses to delivery of juice (red), large picture (blue), and small picture (black) are shown for a single dopamine neuron (left) and population (right, n = number of neurons). Insets at right show response magnitudes (mean ± SEM). Gray areas indicate analysis periods for data shown in inset and in (D)–(F). (B) Blocked trials. The responses to small picture (black) were lower than in pseudorandom trials, whereas juice responses were maintained. (C) Blocked + context trials. Different background pictures indicated separate trial blocks using juice, large pictures, and small pictures, respectively, without liquid spout in picture blocks. Responses to large and small pictures (blue and black) were lower than in pseudorandom and blocked trials. (D–F) Comparisons of normalized neuronal activations between the three contexts. Activations to unpredicted juice (D), large picture (E), and small picture (F) were normalized to baseline activity (set to 1, black horizontal lines) and plotted for individual dopamine neurons (dots) in pseudorandom trials (“P,” left in D–F), blocked trials (“B,” middle), and blocked + context trials (“B+C,” right). Tops and bottoms of gray boxes show 25^th^ and 75^th^ percentiles, respectively; red lines indicate medians. Responses varied significantly between the three stimuli (p < 0.001; F[2,279] = 66.83; two-way ANOVA) and three contexts (p < 0.001; F[2,279] = 21.48; interaction: p = 0.0236; F[4,279] = 3.76). Responses to each stimulus differed individually between contexts (p < 0.05 or p < 0.01; post hoc Scheffé after post hoc one-way ANOVAs: juice: p = 0.002; F[2,91] = 6.68; large picture: p = 0.01; F[2,94] = 4.51; small picture: p < 0.001; F[2,92] = 26.75).

The first test for context dependency involved the presentation of USs at unpredicted times. The juice US in pseudorandom trials elicited typical activations in electrophysiologically characterized dopamine neurons in substantia nigra and ventral tegmental area (see Note S1 and Figure S1 available online) (30 of 33 neurons, 91%; Figure 2A). In the same reward context, the large and small unpredicted pictures induced substantial but shorter dopamine activations (18 and 14 of 33 neurons, 55% and 42%). The picture responses seemed to replicate straightforward dopamine activations to salient stimuli [14, 15, 16, 17]. However, the activations failed to vary with picture size (Figure 2A, blue versus black, inset), were at odds with the reported response absence to novel small pictures before learning [18, 19], and would not reflect motivational salience of the valueless pictures (Figure 1H).

To test possible influences of reward context on reward sensitive dopamine neurons, we used blocked trials separating juice, large picture, and small picture. Whereas juice activations remained strong (22 of 31 neurons, 71%), the activations to the large and small pictures decreased and varied with picture size (10 and 3 of 31 neurons, 32% and 10%; Figure 2B), suggesting context sensitivity. With further context separation in blocked + context trials, the juice activations remained robust (25 of 31 neurons, 81%), whereas the activations to the large picture decreased even more (7 of 31 neurons, 23%) and the activations to the small picture basically disappeared (1 of 31 neurons, 3%; Figure 2C).

Quantitative analyses revealed that all three US activations failed to habituate across successive trials (Note S2; Figures S2A and S2B). Whereas juice responses varied unsystematically (Figure 2D), the activations to both unrewarded pictures decreased monotonically with increasing separation from rewarding contexts (Figures 2E and 2F; p < 0.05–0.01, Scheffé test; after p < 0.01–0.001, one-way ANOVA; after p < 0.001, two-way ANOVA), which enhanced neuronal discrimination (small picture versus juice; Note S3; Figures S2C and S2D). Eye positions during the 500 ms preceding the US revealed indiscriminate focus on the center of the monitor in pseudorandom trials, slightly less central focus in all blocked trials, and very little focus in all blocked + context trials (Figures S2E–S2G). Dopamine responses to all USs were slightly stronger with central fixation (“eyes in” within 5.0 degrees of visual angle) compared to the eyes being off-center (“eyes out”) (Figures S2H–S2K), confirming reported low fixation sensitivity [20]. The fixation effects were indiscriminate in all three contexts and failed to explain the monotonic picture response decreases with increasing separation from reward context (compare Figures 2E and 2F with Figures S2H–S2K).

### Neuronal Responses to Conditioned Stimuli

**Figure 3:**
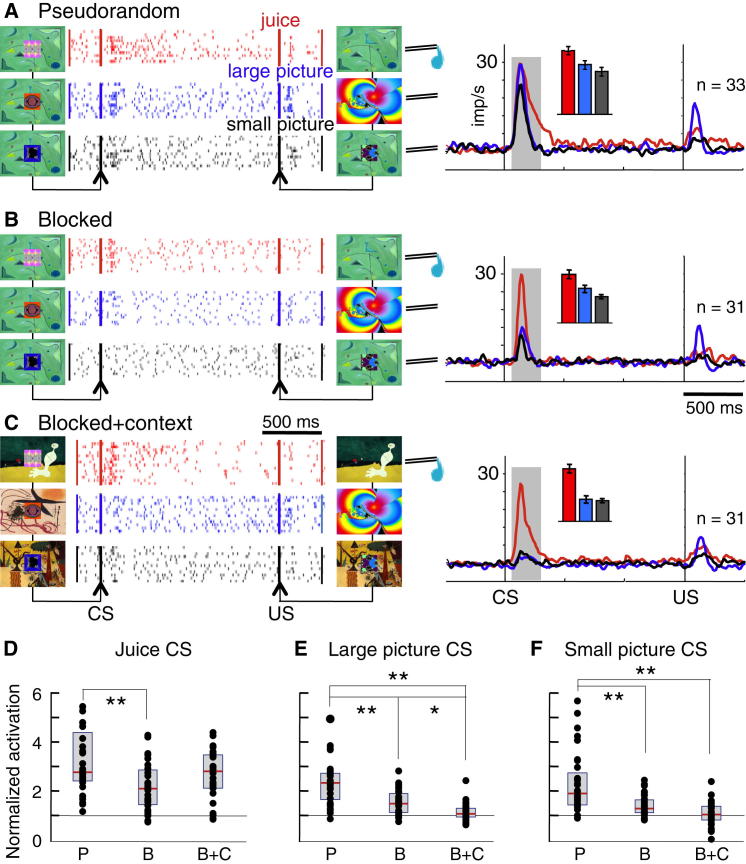
Effects of Context Separation on Dopamine Responses to Conditioned Stimuli Predicting Juice and Pictures (A) Pseudorandom trials. CS and US responses of a single dopamine neuron (left) and population of 33 dopamine neurons (right, n = number of neurons) with juice (red), large picture (blue), and small picture (black). Insets at right show response magnitudes for the three CSs in the gray analysis windows (mean ± SEM). Average CS response latency was 85.4 ± 42.9 ms (mean ± SEM). Durations of responses to juice CS exceeded those to picture CSs, the difference starting at 210.1 ± 123.1 ms after CS onset. (B) Blocked trials. The responses to large and small picture CSs (blue, black) were lower than in pseudorandom trials, whereas juice CS responses were maintained. (C) Blocked + context trials. The CSs for the large and small pictures failed to elicit neuronal responses (blue, black), whereas juice CS responses remained unaltered (red). (D–F) Comparisons of normalized neuronal activations between the three contexts. Same format as for Figures 2D–2F. CS responses varied significantly between the three stimuli (p < 0.001; F[2,281] = 41.42; two-way ANOVA) and three contexts (p < 0.001; F[2,281] = 33.91; interaction: p = 0.114; F[4,281] = 1.88). CS responses for each stimulus differed individually between contexts (p < 0.05 or p < 0.01; post hoc Scheffé after post hoc one-way ANOVAs: juice: p = 0.003; F[2,92] = 6.35; large picture: p = 0.001; F[2,94] = 30.36; small picture: p < 0.001; F[2,93] = 20.23). Not shown: responses to the predicted stimuli (US) differed between the three stimuli (p < 0.001; F[2,281] = 8.74), but not between predicted juice and predicted pictures (p > 0.05; Scheffé). US responses varied inconsistently between the three contexts (p < 0.01; F[2,281] = 4.94; interaction: p = 0.73; F[4,281] = 0.51; two-way ANOVA; p > 0.05 in post hoc Scheffé after post hoc one-way ANOVAs: juice: p = 0.22; F[2,92] = 1.54; large picture: p = 0.05; F[2,94] = 3.06; small picture: p < 0.13; F[2,93] = 2.07).

Would reward context also affect the known dopamine activations to conditioned stimuli (CSs) predicting juice and pictures? In pseudorandom trials, dopamine neurons showed strong activations to the juice CS (26 of 33 neurons, 79%) and slightly weaker activations to the CSs for the large and small pictures (18 and 12 of 33 neurons, 55% and 36%; Figure 3A). Activation peaks were similar for all three CSs. In blocked trials, CS activations remained strong for juice but dropped for both pictures (20, 8, and 2 of 31 neurons, 65%, 26%, and 6%; Figure 3B). In blocked + context trials, juice CS activations remained robust (23 of 31 neurons, 74%), whereas CS activations for both pictures were almost entirely lost (1 of 31 neurons each; Figure 3C). With the predicted USs, activations were highest with the large picture and varied insignificantly between the three contexts (Figures 3A–3C; p > 0.05, Scheffé after ANOVAs).

Further analyses showed no habituation across successive trials with all CS responses (Note S2; Figures S3A and S3B). Responses varied unsystematically for juice CS between contexts (Figure 3D) but decreased monotonically for both picture CSs (Figures 3E and 3F; p < 0.05–0.01, Scheffé test; after p < 0.001, one-way ANOVA; after p < 0.001, two-way ANOVA), which enhanced neuronal discrimination (small picture CS versus juice CS; Note S3; Figures S3C and S3D). The eyes focused indiscriminately on all CSs in pseudorandom trials and somewhat less in blocked trials but lost focus on pictures CSs in blocked + context trials (Figures S3E–S3G). Ocular fixation of the CSs enhanced slightly all dopamine responses irrespective of context (Figures S3H–S3K, “eyes in” versus “eyes out”). Importantly, the CSs for the unrewarded pictures had little effect on dopamine neurons in blocked + context trials, even when the eyes fixated the CSs (Figure S3K), whereas all juice CS responses remained strong without fixation. Thus, eye fixation effects failed to parallel the monotonic picture response decreases with decreasing reward contexts.

### Prediction Errors

**Figure 4:**
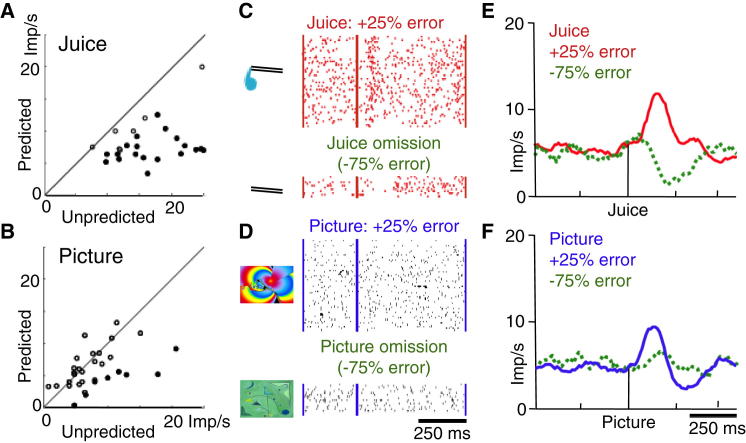
Dopamine Prediction Error Responses with Juice and Picture (A) Comparison between responses of individual dopamine neurons to unpredicted juice without preceding CS (abscissa) and juice predicted by CS (ordinate) in pseudorandom trials (comparing data shown in Figures 2A versus 3A). Filled circles represent significantly higher responses to unpredicted than to predicted juice (p < 0.05, t test); open circles represent insignificant differences. (B) Same as (A), but for unpredicted versus predicted large picture. The smaller differences compared to (A) were still significant in the sampled population (n = 33, p < 0.001; paired t test). (C) Rastergrams of responses from a single dopamine neuron tested with positive (+25% error) and negative (omission: −75% error) juice prediction errors at time of juice in the probabilistic outcome task. (D) Same neuron as (C), but tested with large picture. (E) Population responses to positive and negative juice prediction errors in the probabilistic outcome task (n = 14 neurons). (F) Same as (E), but for picture prediction errors.

Dopamine neurons failed to show negative prediction error responses to the unrewarded picture USs following the respective CSs (Figures 3A–3C). Furthermore, whereas most activations to unpredicted juice exceeded those to predicted juice, only a few activations to the large picture varied significantly with prediction (Figures 4A, 4B, S4A, and S4B). Apparently the neurons were not processing predictions from the unrewarded picture CSs when the picture USs were presented.

To more explicitly test prediction errors, we presented the juice and large-picture CSs to predict respective outcome delivery in 75% and omission in 25% of trials, using pseudorandom alternation and the same background picture as in pseudorandom trials. Outcome delivery would produce a positive, 25% prediction error, and omission would constitute a negative, 75% prediction error. Dopamine neurons showed activations to juice and picture delivery, and depressions to juice omission but not to picture omission (Figures 4A–4D). Thus, dopamine picture activations were moderately affected by positive prediction errors but failed to show clear depressions with negative prediction errors.

## Discussion

These data demonstrate that dopamine neurons are activated by unrewarded stimuli in rewarded environments. Increasing separation between rewarded and unrewarded environments decreased these activations in a graded fashion. These context effects reflected pseudoconditioning and possibly higher-order context conditioning but were not explained by higher-order conditioning to punctate stimuli, stimulus-reward pairing, habituation, eye position, trace conditioning, or sensitization. Although physical salience, novelty, and generalization are known to affect dopamine responses, they failed to explain the current context effect. Thus, reward context constitutes a separate, additional factor affecting dopamine neurons. The mechanism allows rapid reactions to unrewarded stimuli in rewarding contexts, which permits quick encounters with large ranges of potentially rewarding objects. It is readily conceptualized into the overall reward relatedness of the phasic dopamine signal and is evolutionarily adaptive.

The small unpredicted picture failed to induce dopamine responses in the well-separated blocked + context trials but elicited responses in pseudorandom trials (Figure 2). The unrewarded pictures were uncorrelated with juice reward in pseudorandom trials, but the overall reward probability of p = 0.33 would allow Pavlovian reward conditioning of the contextual background, whereas the unrewarded blocks constituted lesser rewarded contexts. Thus, the higher dopamine activations in pseudorandom trials compared to unrewarded blocks likely reflect pseudoconditioning as defined by animal learning theory [6]. The remaining responses to the large picture in the well-separated context might reflect the dopamine sensitivity to sensory impact [21]. This interpretation might also explain variations of dopamine responses to unrewarded stimuli with contextual reward frequency [18, 19] (Figure S4C). Alternatively, the picture USs and CSs may derive their efficacy from higher-order conditioning to the common contextual background picture in pseudorandom and blocked trials, which itself predicts reward with probability of p = 0.33 in pseudorandom trials but no reward in unrewarded blocked trials. The higher-order conditioning would not involve punctate CSs, because these were absent in US-only trials. Thus, the contextual reward influences on dopamine responses to unrewarded stimuli may involve pseudoconditioning or higher-order context conditioning.

Alternatives to context dependency are unlikely to explain the dopamine responses to unrewarded pictures. Explicit delay conditioning was ruled out by the lack of direct pairing with reward. Trace conditioning of unrewarded pictures would be counteracted by the prediction of reward absence against reward probability of p = 0.33 in pseudorandom trials and would have produced weakest rather than strongest activations of all contexts. Physical salience was constant with the identical stimuli in all contexts. Stimulus novelty was low and constant after thousands of trials. Habituation provided no explanation, as the responses failed to decrease with stimulus repetition. Eye position affected nondifferentially all contexts, and only mildly. Response generalization between picture and juice USs was unlikely due to their different sensory modalities. Sensitization assumes existing, intrinsic responses to stimuli and nonassociative stimulus repetition rather than context associations [6]. However, the small picture failed to elicit responses in the unrewarded context, as seen previously [18, 19], and responses were higher in more rewarded contexts and lower with stereotyped stimulus repetition in blocked trials. Thus, contextual influences seem to provide the most coherent explanations.

The moderate positive and negligible negative prediction error responses with pictures contrasted with bidirectional prediction error coding with reward. The absence of bidirectional reward prediction error responses confirms the behaviorally assessed unrewarded nature of the current pictures. Similarly, dopamine neurons fail to code bidirectional prediction errors with aversive stimuli [22, 23], which act via sensory impact in rewarded contexts rather than punishment [21, 24]. Specifically, the absence of negative prediction error responses at unrewarded USs suggests absent reward prediction by the picture CSs. Thus, the poor prediction error coding with the pictures confirms their unrewarded nature and suggests that their responses derive from rewarded contexts.

The context dependency of dopamine responses might explain why dopamine activations are apparently incompatible with straightforward reward coding. It adds to other mechanisms by which unrewarded stimuli elicit or enhance dopamine activations, namely sensory impact [21], generalization [18, 19, 20, 25, 26], and novelty [16, 26]. These activations detect the stimulus before identifying its rewarding nature. These activations might reflect an initial assumption that any stimulus in a rewarded environment could be a reward. Lack of recognition of the modifiable sensory preidentification response led to the assumption of aversive dopamine coding [22, 23, 25]. This preidentification response is followed by a second, stronger component that accurately codes reward value (as prediction error) (Figure S4D). Experiments using random dot motion separate well the two response components [27]. Thus, the influence of rewarded contexts confirms the exquisite reward sensitivity of dopamine neurons [24].

Given the pronounced influence of dopamine activations on approach behavior [28, 29], the influence of context, generalization, and novelty on dopamine responses conceivably facilitates behavioral reactions to potential reward. Such a mechanism would prevent premature asymptotes in reward detection, enhance the pursuit of objects with faint chances of being reward, and minimize reward misses. In addition, the chance to try out such an object and experience its potential reward value would increase if it were detected early on, even before its full identification. This is what the early, preidentification dopamine response might be mediating. Although neuronal discrimination is imperfect at this stage, the very short latency would facilitate early initiation of behavior while leaving time for correction before carrying out the behavior. If the object is indeed a reward, rapid behavioral initiation would result in arriving a bit earlier at the reward than competitors without that detection system and lead to more successful reward encounters. In the long run of evolution, such small advantages enhance fitness [30]. Taken together, the extended responsiveness of dopamine neurons in rewarded contexts may constitute a mechanism that enhances reward detection and may inform theories of optimal reward acquisition.

## Experimental Procedures

### Animals and Behavior

Three adult male rhesus monkeys (A–C, *Macaca mulatta*, 8–9 kg) were behaviorally conditioned and implanted aseptically and stereotaxically with a head holder and recording chamber under general anesthesia. All protocols were approved by the UK Home Office.

The unconditioned stimuli (USs) were fruit juice (fixed quantity of 0.1–0.2 ml), large intense picture (duration 500 ms, size 34.6° × 25.9°, luminance 120–140 cd/m^2^), and small weak picture (500 ms, 4.6° × 4.6°, 8–34 cd/m^2^). Mean US interval was 9.5 s (exponential distribution, mean 4.0 s, truncated at 12.0 s, added to constant interval of 5.5 s, approximating flat hazard rate). The fruit juice was delivered by a computer-controlled valve at a spout at the animal’s mouth; the pictures were shown at the center of a computer monitor 450 mm in front of the animal, counterbalanced between animals. The conditioned stimuli (CSs) were three pictures (1.5 s duration, 4.6° × 4.6°, 8–20 cd/m^2^) predicting, respectively, juice, large picture, and small picture at CS offset. Mean CS intertrial interval was 8 s (from CS offset to next CS onset; exponentially distributed, mean 4.0 s, truncated at 12.0 s, added to constant interval of 4.0 s). A common or specific background picture was present during all trial and intertrial intervals (34.6° × 25.9°, 120–140 cd/m^2^). These stimuli were used in pseudorandom trials (animals A and B), blocked trials (animal C), and blocked + context trials (animals A and B) (Figures 1A–1F).

In the choice task (animals A and B), onset of a central spot (1.3°) required ocular fixation for 500 ms (Figure 1G). The fixation spot was then extinguished, and two CSs appeared simultaneously at 10° to the left and right in pseudorandom alternation (4.6° × 4.6°, same CSs as above, except for two newly trained CSs for small and large pictures accompanied by juice; Figures 1H3–1H5). Following saccadic choice within 800 ms, the unchosen CS disappeared and the chosen outcome was delivered 500 ms later together with disappearance of the chosen CS. Trials were aborted on premature fixation breaks or inaccurate saccades, followed by repetition of same trial type.

### Data Acquisition and Analysis

A head holder and recording chamber were stereotaxically and aseptically implanted under general anesthesia before neuronal recordings. Using conventional extracellular recording techniques, we studied the activity of single midbrain dopamine neurons with moveable single tungsten microelectrodes (Note S1). Discharges from neuronal perikarya were amplified, filtered (300 Hz to 2 kHz), and converted into standard digital pulses by an adjustable Schmitt trigger.

During neuronal recordings, we monitored licking with 0.5 ms resolution by tongue interruptions of an infrared optosensor 4 mm below the spout (model V6AP; STM Sensor Technology) and recorded eye position with 5 ms resolution using an infrared eye tracking system (ETL200; ISCAN). We measured visual stimulus intensity with a luminance meter (LS-100; Konica Minolta). Custom software using MATLAB (The MathWorks) and Psychophysics Toolbox [31] served to control behavior and record signals from neurons, stimuli, eye positions, and licking.

Statistical tests served to identify significant neuronal responses in analysis time windows (control period: −500 to 0 ms versus response period: 50–300 ms after US or CS; p < 0.01; paired t test) and to compare neuronal responses between outcomes and contexts (one-way and two-way ANOVA with post hoc Scheffé test).

## Supplemental Information

Document S1. Figures S1–S4 and Notes S1–S3
