# Reward Contexts Extend Dopamine Signals to Unrewarded Stimuli

**DOI:** 10.1016/j.cub.2014.01.057

**Published:** 2014-02-17

**Authors:** Shunsuke Kobayashi, Wolfram Schultz

(Current Biology *24*, 56–62; January 6, 2014)

As a result of an oversight during the production process, Figure S4 in the online supplemental information for this article was erroneously substituted by a duplicate of the main Figure 4. (Only the figure itself was erroneous; the accompanying Figure S4 legend was correct.) The correct Figure S4 is now included in the supplement online. The authors and the journal apologize for any confusion caused by this error.

