# Supplementary material for: Reward Contexts Extend Dopamine Signals to Unrewarded Stimuli
Source: Curr Biol. 2014 Jan 6;24(1):56–62. doi: 10.1016/j.cub.2013.10.061 (PMC3898276; doi:10.1016/j.cub.2013.10.061)
Supplement: Document S1. Figures S1–S4 and Notes S1–S3 [file mmc1.pdf]

Current Biology, Volume *24*

Supplemental Information

Reward Contexts Extend Dopamine Signals to Unrewarded Stimuli

Shunsuke Kobayashi and Wolfram Schultz

Supplemental Inventory

Figure S1, Related to Figure 1
Figure S2, Related to Figure 2
Figure S3, Related to Figure 3
Figure S4, Related to Figure 4
Note S1
Note S2
Note S3
Supplemental References

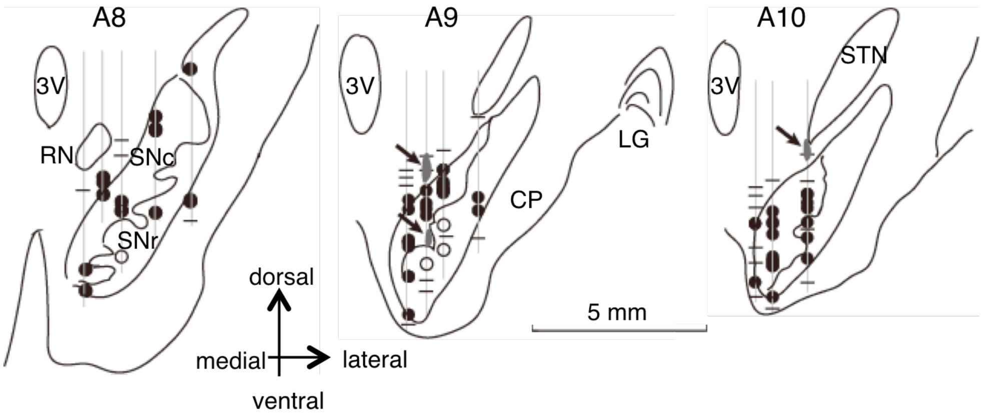

Figure S1, Related to Figure 1. Histological reconstruction of recording positions of dopamine neurons in the ventral midbrain. Approximate anteroposterior levels (A8, A9, A10) are shown in millimeters relative to the interaural line according to an atlas [S2]. Filled circles, recording positions of electrophysiologically characterized putative dopamine neurons. Horizontal lines, nondopamine neurons. Open circles, nondopamine neurons with high discharge frequencies (>30 impulses/sec). Arrows point to electrolytic lesions made after all recordings for identifying recording tracks. Data from animals A and B were collapsed onto representative coronal sections drawn from monkey A. RN, red nucleus; SNc, pars compacta of substantia nigra; SNp, pars reticulata of substantia nigra; 3V, third ventricle; CP, cerebral peduncle; LG, lateral geniculate body.

Figure S2

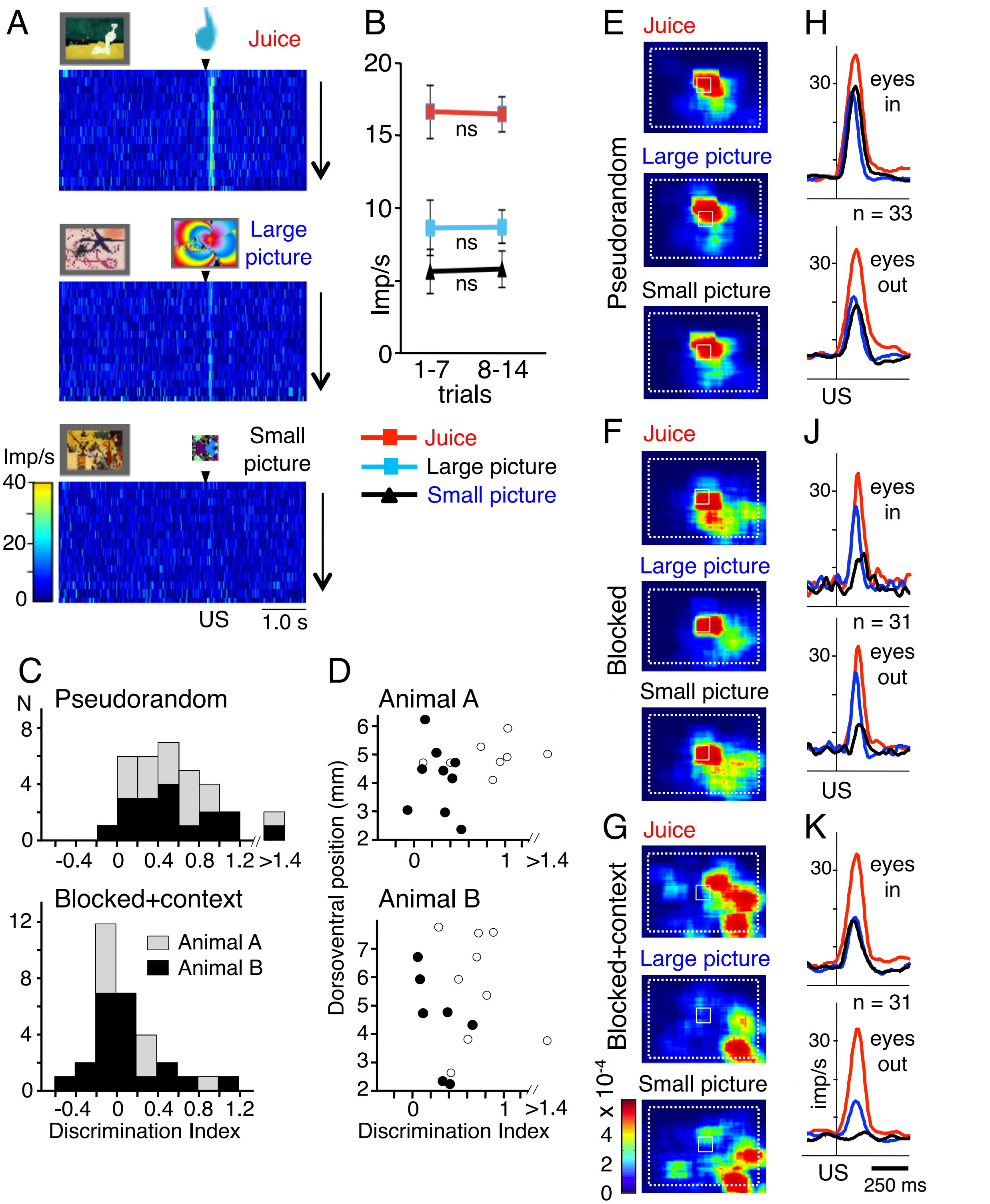

A
Juice
Large picture
Small picture
Imp/s
40
20
0
US
1.0 s
B
20
15
10
5
0
Imp/s
ns
1-7
8-14
trials
Juice
Large picture
Small picture
C
N
Pseudorandom
8
4
0
-0.4
0
0.4
0.8
1.2
>1.4
Blocked+context
12
Animal A
Animal B
Discrimination Index
D
Animal A
Animal B
Dorsoventral position (mm)
Discrimination Index
E
Pseudorandom
F
Blocked
G
Blocked+context
x 10-4
H
J
K
eyes in
eyes out
n = 33
n = 31
30
imp/s
250 ms

Figure S2, Related to Figure 2. Neuronal responses to unpredicted unconditioned stimuli (US): lack of response habituation, increased discrimination and inscriminate influence of ocular fixation
(A) Lack of habituation of dopamine US responses in 'blocked+context' trials. Heat plots show trial-by-trial population activity aligned on juice (top), large picture (middle) and small picture (bottom). Activities in each trial were averaged across 31 dopamine neurons and are displayed as one line of activity as impulses/s according to the colour code shown at bottom left. The three trial types were tested in separate blocks, identified by their specific background picture on the monitor (top left). Trial sequence in each block is shown from top to bottom in each panel (arrows).
(B) Quantification of data shown in A, separating first 14 from next 14 trails. ns: $p>0.1$ (t-test).
(C) Decrease of neuronal discrimination indices for small picture vs. juice from 'pseudorandom' trials (top; n=33 neurons) to 'blocked+context' trials (bottom; n=31 neurons), suggesting higher discrimination with better context separation.
(D) Relationships between recording positions and discrimination indices during 'pseudorandom' trials in animals A and B. Dorsoventral position refers to the horizontal plane through the interaural line according to the atlas by Paxinos [S2]. Each point represents normalized activation from an individual neuron tested with small picture. Filled circles, significantly lower activations to small picture than to juice ($p<0.05$; paired t-test).
(E-G) Influences of reward context on ocular fixations preceding the USs. Top: indiscriminate fixation on monitor center in 'pseudorandom' juice and picture trials, compatible with lack of predictive information about upcoming US. Middle: slightly less focussed fixation with all USs in 'blocked' trials. Bottom: poor eye fixations with all USs in 'blocked+context' trials with highest context separation. Calibration indicates probability of eyes being in central 0.5°x0.5° area during 500 ms immediately preceding US onset (solid white squares; probabilities were Gaussian smoothed, standard deviation = 1.0°). Dotted white lines outline the computer monitor.
(H-K) Influences of eye positions on neuronal US responses. The analysis separated trials with central fixation ('eyes in') from trials without central fixation ('eyes out': outside of central 0.5°x0.5° area).

Figure S3

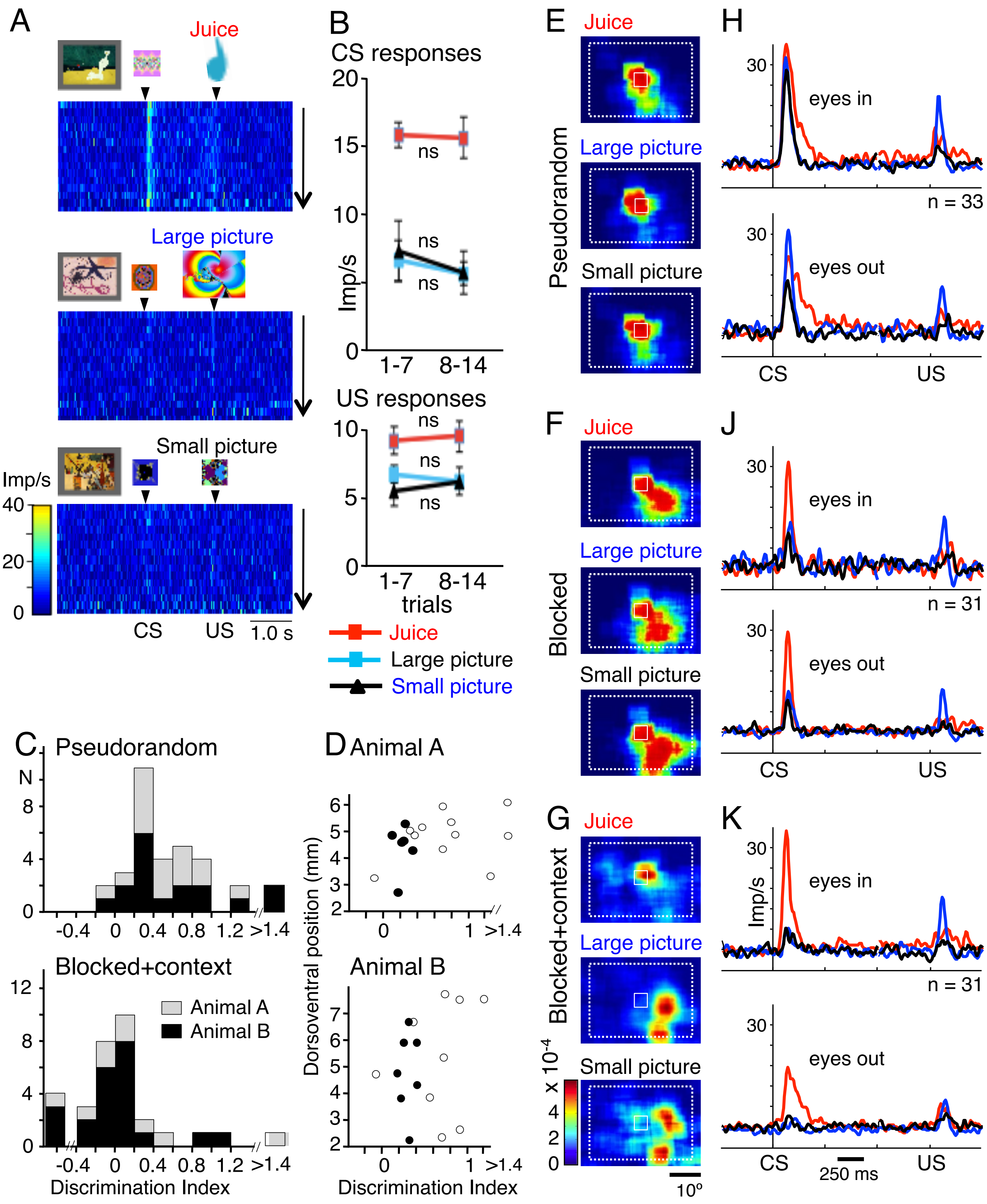
A
Juice
Large picture
Small picture
Imp/s
40
20
0
CS
US
1.0 s
B
CS responses
20
15
10
5
0
ns
Imp/s
1-7
8-14
US responses
10
5
0
trials
Juice
Large picture
Small picture
C
Pseudorandom
N
8
4
0
-0.4
0
0.4
0.8
1.2
>1.4
Blocked+context
12
Animal A
Animal B
Discrimination Index
D
Animal A
Animal B
Dorsoventral position (mm)
Discrimination Index
E
F
G
Pseudorandom
Blocked
Blocked+context
x 10^-4
10°
H
J
K
30
eyes in
eyes out
n = 33
n = 31
Imp/s
250 ms

Figure S3, Related to Figure 3. Neuronal responses to conditioned stimuli (CS): lack of response habituation, increased discrimination and inscriminate influence of ocular fixation (same conventions as for Figure 2)
(A, B) Lack of habituation of dopamine CS responses in 'blocked+context' trials.
(C) Decrease of neuronal discrimination indices for small picture vs. juice from 'pseudorandom' trials (top; n=33 neurons) to 'blocked+context' trials (bottom; n=31 neurons), suggesting higher discrimination with better context separation.
(D) Relationships between recording positions and discrimination indices during 'pseudorandom' trials in animals A and B.
(E-G) Influences of reward context on ocular fixations preceding the CSs.
(H-K) Influences of eye positions on neuronal CS responses. The analysis separated trials with CS fixation from trials without CS fixation. 'Eyes in' and 'eyes out' refer to eye positions within central $0.5^\circ x 0.5^\circ$ area.

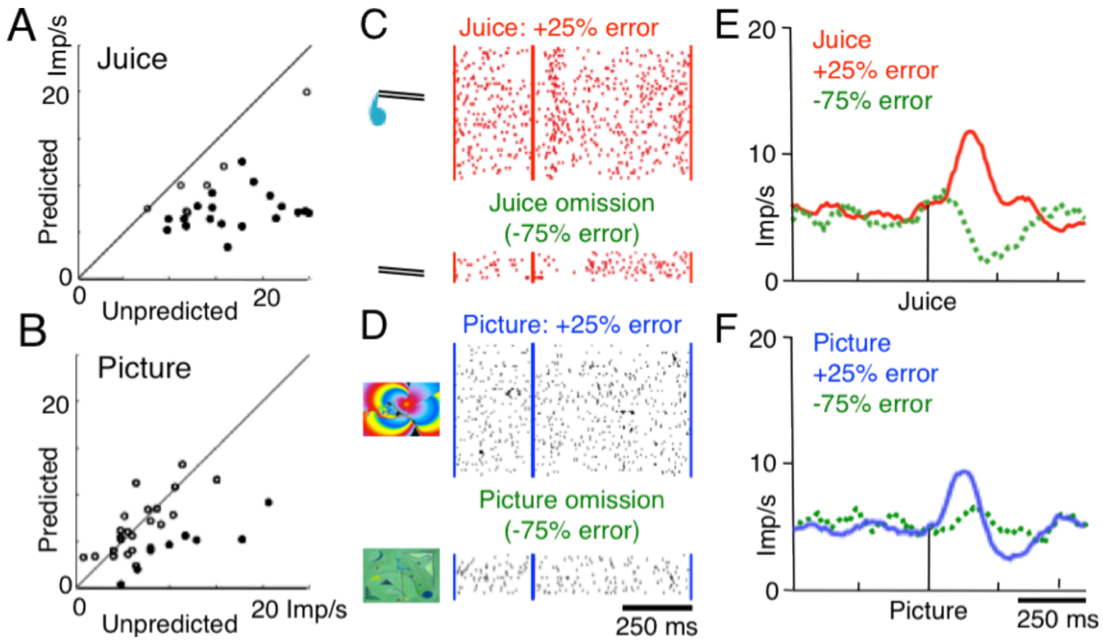

Figure S4, Related to Figure 4. Comparisons of dopamine activations between unpredicted and predicted outcomes, earlier observed reward context dependent dopamine activations, and schematic dopamine activation components

(A) Comparison between responses of individual dopamine neurons to unpredicted juice without preceding CS (abscissa) and juice predicted by CS (ordinate) in 'blocked+context' trials (comparing data shown in Figure 2C vs. 3C). Filled circles, significantly higher responses to unpredicted than predicted juice ($p<0.05$, t-test).

(B) Same as A but for unpredicted vs. predicted large picture in 'blocked+context' trials. The smaller differences compared to A were still significant in the sampled population ($n=31$, $p<0.001$; paired t-test).

(C) Decreasing dopamine activations to unrewarded stimuli with different context reward probability in earlier experiments. Reward predicting stimuli elicited statistically significant activations in 70% of dopamine neurons (left). When three of four stimuli were rewarded, unrewarded stimuli elicited significant activations in 125 of 286 dopamine neurons (44%) (center [S3]). By contrast, when only one of four stimuli was rewarded, unrewarded stimuli activated only 1 of 112 dopamine neurons (right [S4]). The different reward probabilities in these experiments induced specific reward contexts in a similar way as the systematically varied trial schedules did in the current experiments, supporting the suggestion that reward context influences the responsiveness of dopamine neurons.

(D) Schematics of the two dopamine activation components to stimuli (arrow). The initial component (blue) codes sensory stimulus impact and is highly susceptible to reward context, generalization and novelty (double arrow), the second component (red) codes reward value (as prediction error). Graph inspired by Fiorillo et al. [S5].

Note S1: Histological reconstruction of neuronal recording positions
We recorded the activity of single midbrain dopamine neurons with single, custom made, glass insulated, platinum plated tungsten microelectrodes that were moved inside a daily inserted metal guide cannula in the vertically stereotaxic plane. The following standard electrophysiologically features served to attribute activity to a dopamine neuron recorded in vivo: (1) polyphasic, initially positive or negative waveforms followed by prolonged opposite afterpolarisation, (2) waveform durations of 1.8 – 3.6 ms (100 Hz high-pass filter), and (3) irregular, low baseline firing (0.5–8.5 impulses/s) contrasting with more frequently firing substantia nigra pars reticulata neurons [S1]. Neurons meeting the three criteria frequently showed phasic activation after temporally unpredicted drops of fruit juice outside of tasks. Dopamine neurons were functionally localized relative to trigeminal somatosensory thalamic neurons in awake animals and under general anaesthesia (very small perioral and intraoral receptive fields, high proportion of tonic responses, 2-3 mm dorsoventral extent), and tonic, position coding oculomotor neurons and phasic direction coding pre-oculomotor neurons in awake animals.

After all neuronal recordings were completed, animals A and B received an overdose of pentobarbital sodium (90 mg/kg, i.v.), followed by 4% paraformaldehyde in 0.1 M phosphate buffer through the left ventricle of the heart. Frozen sections were cut coronally in the stereotaxic plane at every 50 µm parallel to recording tracks. Positions of neurons in midbrain groups A8, A9 and A10 were reconstructed after tyrosine hydroxylase immunoreaction or cresyl violet staining (Figure S1). Due to ongoing experiments, histological examination has not been done with animal C.

Note S2: Lack of response habituation
The differences in dopamine activations to the pictures between the differently rewarded contexts might have been due to habituation across successive trials. To assess this possibiliy, we plotted the dopamine responses across individual 'blocked+context' trials for unpredicted USs and CSs and compared the responses between the first seven and the next seven trials in a given block (Figures S2B, S3B). With unpredicted USs, not only the robust juice responses but also the weaker large picture responses persisted unchanged throughout the trial blocks (Figure S2A, B; $p>0.1$; t-test). With the CSs, only the juice CS elicited responses, whereas the picture CSs failed to induce appreciable responses (Figure S3A, B). None of these responses were stronger in the first trial of a given block compared to the following trials ($p>0.1$; t-test). The absence of habituation suggested that the surprise generated by the onset of the first unpredicted US or CS in a new trial block failed to affect the activations of dopamine neurons. (With block designs, habituation refers to the surprise generated across successive trials, between the last trial of the previous block and the first trial of a new block, as opposed to lack of surprise across consecutive trials of the same, known nature. By contrast, the prediction error generated by the temporally unpredicted onset of an event reflects surprise within a trial.)

Note S3: Increasing event discrimination with separation from rewarding context
The decreasing neuronal activations to unrewarded pictures compared to juice suggested that context separation improved event discrimination. To quantify these effects, we calculated a normalized discrimination index between the juice and the small unrewarded picture and then compared that index between 'pseudorandom' and 'blocked+context' trials. We used the count of impulses/s in the analysis time windows after the juice and the small picture, respectively, and subtracted baseline activity before the respective events. The neuronal discrimination index for the unpredicted outcomes (US) was defined as USDI = (activity after small picture – baseline) / (activity after juice – baseline). In analogy, the discrimination index for CS (CSDI) used activity following the respective CS while subtracting baseline activity before each CS. Both DIs were inversely related to discrimination; a DI=1 indicated

no discrimination, and a DI<1 indicated lower activation to the small picture or its CS as compared to juice or its CS.

The USDIs for activations to unpredicted juice vs. small picture were significantly higher during 'pseudorandom' trials compared to 'blocked+context' trials (Figure S2C). DIs were 0.56±0.11 (mean±standard error of the mean, SEM) for both animals in 'pseudorandom' trials and 0.09±0.07 and 0.14±0.11 for 'blocked+context' trials in animals A and B, respectively ($p<0.0001$ between 'pseudorandom' and 'blocked+context', $p>0.2$ between animals; 2-way Anova). Thus event discrimination improved significantly with better context separation. The USDIs failed to correlate with vertical, mediolateral and anteroposterior positions of the recorded neurons (Figure S2D; $p>0.1$; Pearson).

Similarly, the CSDIs for activations to the conditioned stimuli for juice vs. small picture were significantly higher during 'pseudorandom' trials compared to 'blocked+context' trials (Figure S2C). DIs were 0.61±0.13 (mean±SEM) and 0.51±0.09 for 'pseudorandom' trials in animals A and B, respectively, and -0.01+0.09 and 0.17+0.22 for 'blocked+context' trials in animals A and B, respectively ($p<0.0001$ between 'pseudorandom' and 'blocked+context', $p>0.3$ between animals; 2-way Anova). Thus, as with the US activations, event discrimination improved significantly with better context separation. The CSDIs failed to correlate with vertical, mediolateral and anteroposterior positions of the recorded neurons (Figure S3D; $p>0.1$; Pearson).

Supplemental References

S1. Schultz, W., and Romo, R. (1987). Responses of nigrostriatal dopamine neurons to high intensity somatosensory stimulation in the anesthetized monkey. J. Neurophysiol. *57*, 201-217.

S2. Paxinos, G., Huang, X.-F., and Toga, A.W. (2000). The Rhesus Monkey Brain in Stereotaxic Coordinates (Academic Press: San Diego).

S3. Waelti, P., Dickinson, A., and Schultz, W. (2001). Dopamine responses comply with basic assumptions of formal learning theory. Nature *412*, 43-48.

S4. Tobler, P.N., Dickinson, A., and Schultz, W. (2003). Coding of predicted reward omission by dopamine neurons in a conditioned inhibition paradigm. J. Neurosci. *23*, 10402-10410.

S5. Fiorillo, C.D., Song, M.R., and Yun, S.R. (1990). Multiphasic temporal dynamics in responses of midbrain dopamine neurons to appetitive and aversive stimuli. J. Neurosci. **33**, 4710–4725 (2013).
